# Network Analysis of the Multidimensional Symptom Experience of Oncology

**DOI:** 10.1038/s41598-018-36973-1

**Published:** 2019-02-19

**Authors:** Nikolaos Papachristou, Payam Barnaghi, Bruce Cooper, Kord M. Kober, Roma Maguire, Steven M. Paul, Marilyn Hammer, Fay Wright, Jo Armes, Eileen P. Furlong, Lisa McCann, Yvette P. Conley, Elisabeth Patiraki, Stylianos Katsaragakis, Jon D. Levine, Christine Miaskowski

**Affiliations:** 1 Centre for Vision, Speech and Signal Processing, University of Surrey, Guildford, UK; 2 University of California, San Francisco, USA; 3 University of Strathclyde, Glasgow, Scotland; 4 Department of Nursing, Mount Sinai Medical Center, New York, USA; 5 School of Nursing, Yale University, New Haven, USA; 6 School of Nursing, Midwifery and Health Systems, University College Dublin, Dublin, Ireland; 7 School of Nursing, University of Pittsburgh, Pittsburgh, USA; 8 National and Kapodistrian University of Athens, Athens, Greece; 9 Faculty of Nursing, University of Peloponnese, Sparti, Greece; 10 School of Health Sciences, University of Surrey, Guildford, UK

## Abstract

Oncology patients undergoing cancer treatment experience an average of fifteen unrelieved symptoms that are highly variable in both their severity and distress. Recent advances in Network Analysis (NA) provide a novel approach to gain insights into the complex nature of co-occurring symptoms and symptom clusters and identify core symptoms. We present findings from the first study that used NA to examine the relationships among 38 common symptoms in a large sample of oncology patients undergoing chemotherapy. Using two different models of Pairwise Markov Random Fields (PMRF), we examined the nature and structure of interactions for three different dimensions of patients’ symptom experience (i.e., occurrence, severity, distress). Findings from this study provide the first direct evidence that the connections between and among symptoms differ depending on the symptom dimension used to create the network. Based on an evaluation of the centrality indices, nausea appears to be a structurally important node in all three networks. Our findings can be used to guide the development of symptom management interventions based on the identification of core symptoms and symptom clusters within a network.

## Introduction

Oncology patients undergoing cancer treatment experience an average of fifteen unrelieved symptoms that are highly variable in both their severity and distress^1–3^. In order to advance symptom management science and gain a better understanding of oncology patients’ symptom experiences, research has focused on the evaluation of symptom clusters using techniques such as exploratory factor analysis or cluster analysis^4–6^. One of the underlying assumptions of this research is that symptoms that cluster together may share underlying mechanisms that are potential targets for therapeutic interventions. While progress is being made in symptom clusters research^4^, one of the major gaps in knowledge using standard statistical approaches is that the nature of the relationships among individual symptoms and symptom clusters have not been evaluated. This gap in knowledge prevents the identification of key symptom(s) that exert an influence on other co-occurring symptoms or symptom clusters that may be potential target(s) for therapeutic interventions. In this study, we investigate the application of Network Analysis (NA) methods to better understand and interpret the associations among co-occurring symptoms and symptom clusters in oncology patients receiving chemotherapy (CTX).

NA^7–9^ is a graph theory based methodology that is being used to gain new insights into systems biology^10,11^ depression^12,13^, post-traumatic stress^14^, complex bereavement^15^, quality of life (QOL)^16^, and identifying high-risk cancer sub-population^17^. In terms of oncology patients, NA allows one to visualize and interpret quantitatively the relationships among various symptoms and symptom clusters that patients are experiencing. While NA is being used to understand the associations among psychiatric symptoms^18–22^ and substance abuse and dependence symptoms^23^, only one study was found that used NA to evaluate symptoms in oncology patients^24^. Using data on the occurrence of 18 symptoms in 665 oncology patients, a force directed layout algorithm was used to visualize a patient-symptom bipartite network. Then four quantitative methods were used to analyse the patterns of symptom occurrence suggested by the network visualizations. The authors concluded that cancer symptoms occur in a nested pattern as opposed to distinct clusters^24^.

While a historic study^24^, the conclusions regarding the absence of distinct symptom clusters warrants additional exploration because of the limitations and associated implications of the NA methods that were used. For example, modularity optimization has a resolution limit that may prevent it from detecting clusters which are comparatively small with respect to the graph as a whole, even when they are well defined communities^25^. In addition, during unweighted or weighted one-mode projection, some information is lost and the final models do not hold the complete structural information of bipartite networks^26^. As mentioned by the authors^24^, their methods concealed how the groups of symptoms co-occurred, as well as their globally optimal co-occurrence frequencies. In the current study, we explore the complex organisation and interconnectedness of cancer symptoms and associated clusters by using two different models of Pairwise Markov Random Fields (PMRF)^27–29^ on binary symptom occurrence and ordinal symptom severity and distress data.

As part of a symptom assessment, oncology patients are asked to rate not only the occurrence of the symptom, but its associated severity and distress^30–33^. Two of the unanswered questions in symptom clusters’ research is whether the number and types of symptom clusters differ based on the dimension used to create the cluster and how symptoms within and across clusters are related to each other^4,5^. Our study is the first to use NA to evaluate the relationships among symptoms and symptom clusters using ratings of symptom occurrence, severity, and distress, in a sample of oncology patients undergoing chemotherapy (CTX; n = 1328). We used NA to examine the relationships among 38 common symptoms and to explore if the network structures for occurrence, severity, and distress have different properties. Our analyses show the prevalence, importance, and influence of each symptom within each network and the overall connectivity of cancer symptoms within each symptom dimension network. In addition, the interrelationships among symptoms inside and outside of a symptom cluster are described.

## Material and Methods

### Patients and Settings

This secondary analysis is part of a longitudinal study of the symptom experience of oncology outpatients receiving CTX. The methods for this study are described in detail in our previous publications^34–36^. For this NA, enrollment assessment data from the parent, longitudinal study were analysed (n = 1328). Patients were eligible to participate if they: were ≥18 years of age; had a diagnosis of breast, gastrointestinal (GI), gynecological (GYN), or lung cancer; had received CTX within the preceding four weeks; were scheduled to receive at least two additional cycles of CTX; were able to read, write, and understand English; and gave written informed consent. Patients were recruited from two Comprehensive Cancer Centers, one Veteran’s Affairs hospital, and four community-based oncology programs. This study was approved by the Committee on Human Research at the University of California, San Francisco. All methods were performed in accordance with the relevant guidelines and regulations. A written informed consent was obtained from all patients.

### Cancer Symptom Dimensions

A modified version of the Memorial Symptom Assessment Scale (MSAS)^33^ was used to evaluate the occurrence, severity, and distress of 38 symptoms commonly associated with cancer and its treatment. In addition to the original 32 MSAS symptoms, the following six symptoms were assessed: hot flashes, chest tightness, difficulty breathing, abdominal cramps, increased appetite, and weight gain. The MSAS is a self-report questionnaire designed to measure the multidimensional experience of symptoms. Using the MSAS, patients were asked to indicate whether or not they had experienced each symptom in the past week (i.e., symptom occurrence). If they had experienced the symptom, they were asked to rate its severity and distress. Symptom severity was measured using a 4-point Likert scale (i.e., 1 = slight, 2 = moderate, 3 = severe, 4 = very severe). Symptom distress was measured using a 5-point Likert scale (i.e., 0 = not at all, 1 = a little bit, 2 = somewhat, 3 = quite a bit, 4 = very much). The reliability and validity of the MSAS are well established in studies of oncology inpatients and outpatients^33^.

### Network analysis

**Figure 1:**
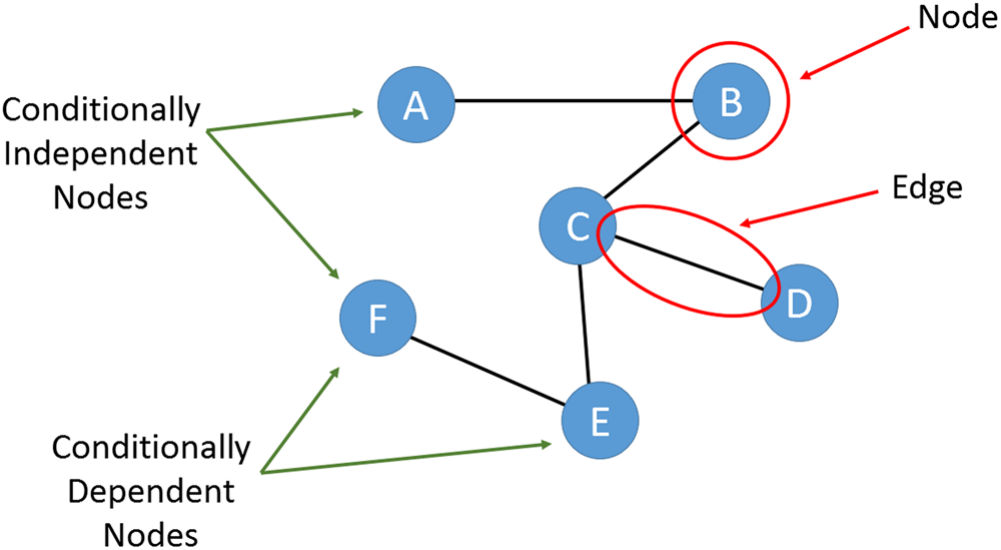
A Pairwise Markov Random Field (PMRF) or an undirected graphical model with 6 nodes, A to F. The presence of edges between nodes indicates the conditional dependency between them.

In general, networks are defined as a collection of interconnected components (i.e., in this paper, symptoms). These components are called nodes and their interaction links are called edges^37^. A Pairwise Markov Random Field (PMRF)^29^ is an undirected graphical model of a set of random variables having a Markov property, described by this undirected graph (or network). Its edges indicate the full conditional association between two nodes after conditioning on all of the other nodes in the network. When a relationship exists between two nodes (i.e., symptoms) that cannot be explained by any other node in the network, these two nodes are connected. The absence of an edge between two nodes (i.e., symptoms) indicates that these nodes are conditionally independent of each other given the other nodes in the network (Fig. 1).

When estimating a PMRF, the number of parameters that need to be estimated grows quickly with the size of the network^38^. In our 38-node networks, 741 parameters (i.e., 38 threshold parameters and 38 × 37/2 = 703 pairwise association parameters) needed to be estimated^38^. To estimate this number of parameters in a reliable fashion, the number of observations in our sample needed to be at least equivalent, which it was given a sample size of 1328 patients.

To create the networks, we used the generalization of the Ising model presented in the IsingFit R-package^39^ for the occurrence data and the polychoric correlation method^28^ for the severity and distress data, using the R-package qgraph^40^. Both approaches entailed the application of a statistical regularization technique, which provided an extra penalty for model complexity. The edges that were likely to be spurious or false positives were removed from the models, leading to networks that were more interpretable.

The model used in the IsingFit R-package^39^ is a binary equivalent of the Gaussian approximation method. Its variables can have only two states and interactions are considered pairwise. The aforementioned model contains two node-specific parameters: the interaction parameter *β*_*jk*_, representing the strength of the interaction between variable j and k, and the node parameter *τ*_*j*_, which represents the autonomous disposition of the variable to take the value of one - “1” - regardless of neighboring variables. The IsingFit model estimates the aforementioned parameters using logistic regression. Through repetition, every variable is regressed on all of the other variables. To obtain sparsity, an ${\ell }_{1}$-penalty is imposed on the regression coefficients. The level of shrinkage depends on the penalty parameter of the lasso. In the IsingFit method, the Extended Bayesian Information Criterion (EBIC) is used to select the set of neighbor nodes that yield the lowest EBIC and in this way constructs the final “true” network.

By viewing X_*j*_ as the response variable and all the other variables X_\*j*_ as the predictors, the EBIC is represented as:

1 $$BI{C}_{\gamma }(j)=-\,2\ell ({\hat{{\rm{\Theta }}}}_{j})+|J|\cdot \,\mathrm{log}(n)+2\gamma |J|\cdot \,\mathrm{log}(p-1)$$

in which $\ell ({\hat{{\rm{\Theta }}}}_{{\rm{j}}})$ is the log likelihood of the conditional probability of X_*j*_ given its neighbours, X_*ne*(*j*)_, |J| is the number of neighbours selected by logistic regression at a certain penalty parameter *ρ*, n is the number of observations, *p* − 1 is the number of covariates (predictors), and c is a hyperparameter, determining the strength of prior information on the size of the model space. The model with the set of neighbours J that has the lowest EBIC is selected.

For severity and distress, we used the R-package qgraph^40^ and applied the polychoric correlation method in combination with the graphical “least absolute shrinkage and selection operator” (glasso) algorithm^28,41,42^. The glasso algorithm by inverting its input, which is the sample’s polychoric correlation matrix, returns a sparse network model where only a relatively small number of edges are used to explain the covariance structure in the data. More precisely, the graphical lasso estimator is the $\hat{{\rm{\Theta }}}$ such that:

2 $$\hat{{\rm{\Theta }}}={{\rm{a}}{\rm{r}}{\rm{g}}{\rm{m}}{\rm{i}}{\rm{n}}}_{{\rm{\Theta }}\ge 0}({\rm{t}}{\rm{r}}(S{\rm{\Theta }})-\,{\rm{l}}{\rm{o}}{\rm{g}}\,det({\rm{\Theta }})+\lambda \,\sum _{j\ne k}\,|{{\rm{\Theta }}}_{jk}|)$$

where *S* is the sample’s polychoric correlation matrix, and *λ* is a penalizing parameter. Glasso utilizes this penalizing parameter to control the degree to which regularization is applied. This penalising parameter can be selected by minimizing the EBIC. In general, graphical lasso controls the relationships between the variables in a network and gives partial correlations between variables, which increases the parsimony of the final network models^28,42^.

The above mentioned techniques allowed us to create and construct the networks using the symptom occurrence, severity, and distress data. However, it is crucial to establish robust methods to assess the stability and accuracy of the network. The next section discusses our approach to assess and evaluate the constructed networks.

### Network Assessment

**Figure 2:**
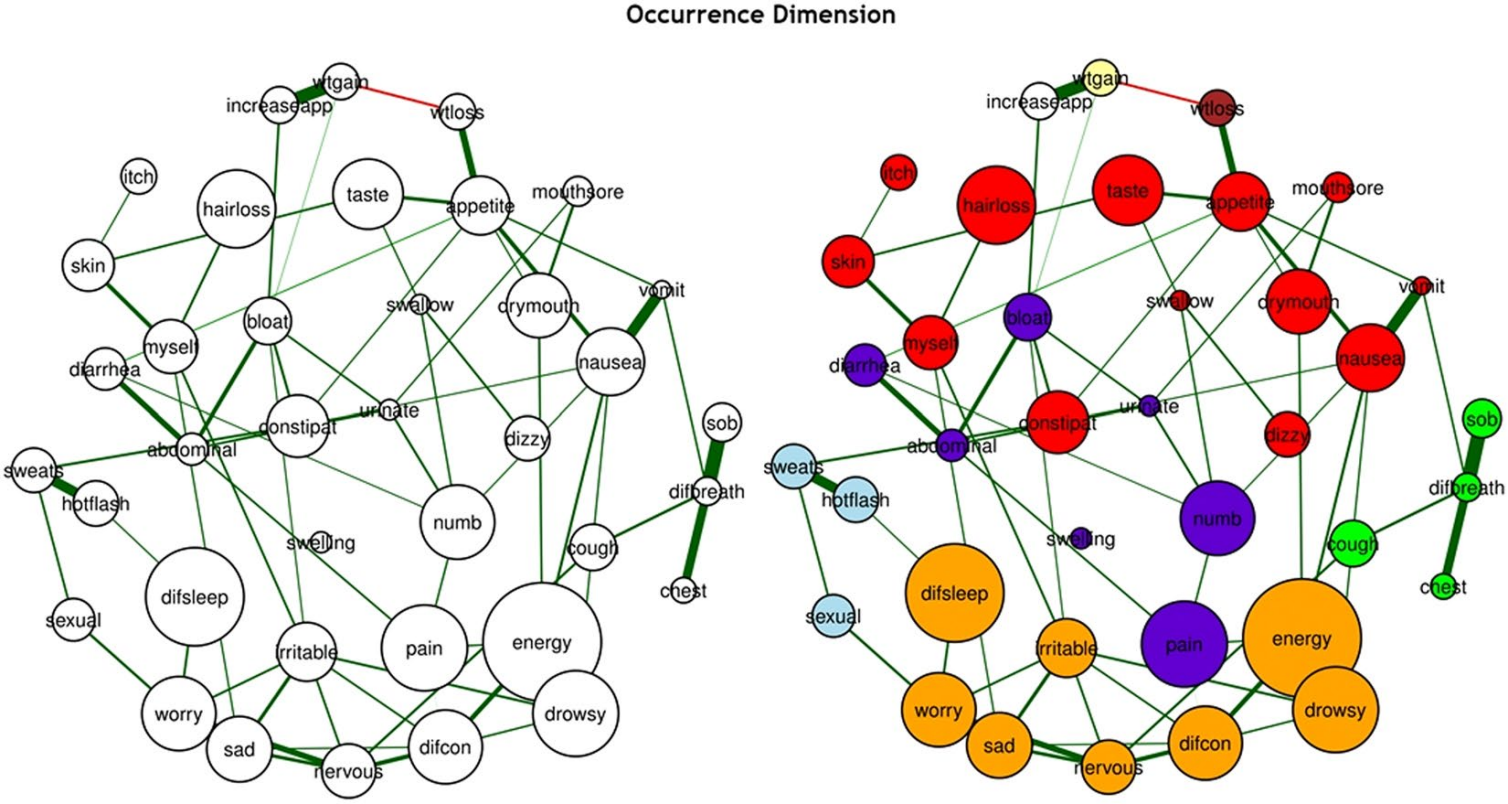
The estimated networks of 38 cancer symptoms across the “occurrence” dimension (a) without the identified communities and (b) with the identified communities (walktrap algorithm). Nodes represent symptoms and edges represent pairwise dependencies between the symptoms, after controlling for all of the other correlations of a given node. The 38 cancer symptoms represented in the nodes above are coded in the following fashion: difcon: Difficulty Concentrating, pain: Pain, energy: Lack of Energy, cough: Cough, nervous: Feeling Nervous, hotflash: Hot Flashes, drymouth: Dry Mouth, nausea: Nausea, drowsy: Feeling Drowsy, numb: Numbness or Tingling in Hands or Feet, chest: Chest Tightness, difbreath: Difficulty Breathing, difsleep: Difficulty Sleeping, bloat: Feeling Bloated, urinate: Problems with Urination, vomit: Vomitting, sob: Shortness of Breath, diarrhea: Diarrhea, sad: Feeling Sad, sweats: Sweats, sexual: Problems with Sexual Interest or Activity, worry: Worrying, itch: Itching, appetite: Lack of Appetite, abdominal: Abdominal Cramps, increaseapp: Increased Appetite, wtgain: Weight Gain, dizzy: Dizziness, swallow: Difficulty Swallowing, irritable: Feeling Irritable, mouthsore: Mouth Sore, wtloss: Weight Loss, hairloss: Hair Loss, constipat: Constipation, swelling: Swelling, taste: Change in the Way Food Tastes, myself: I Do Not Look Like Myself, skin: Changes in Skin.

**Figure 3:**
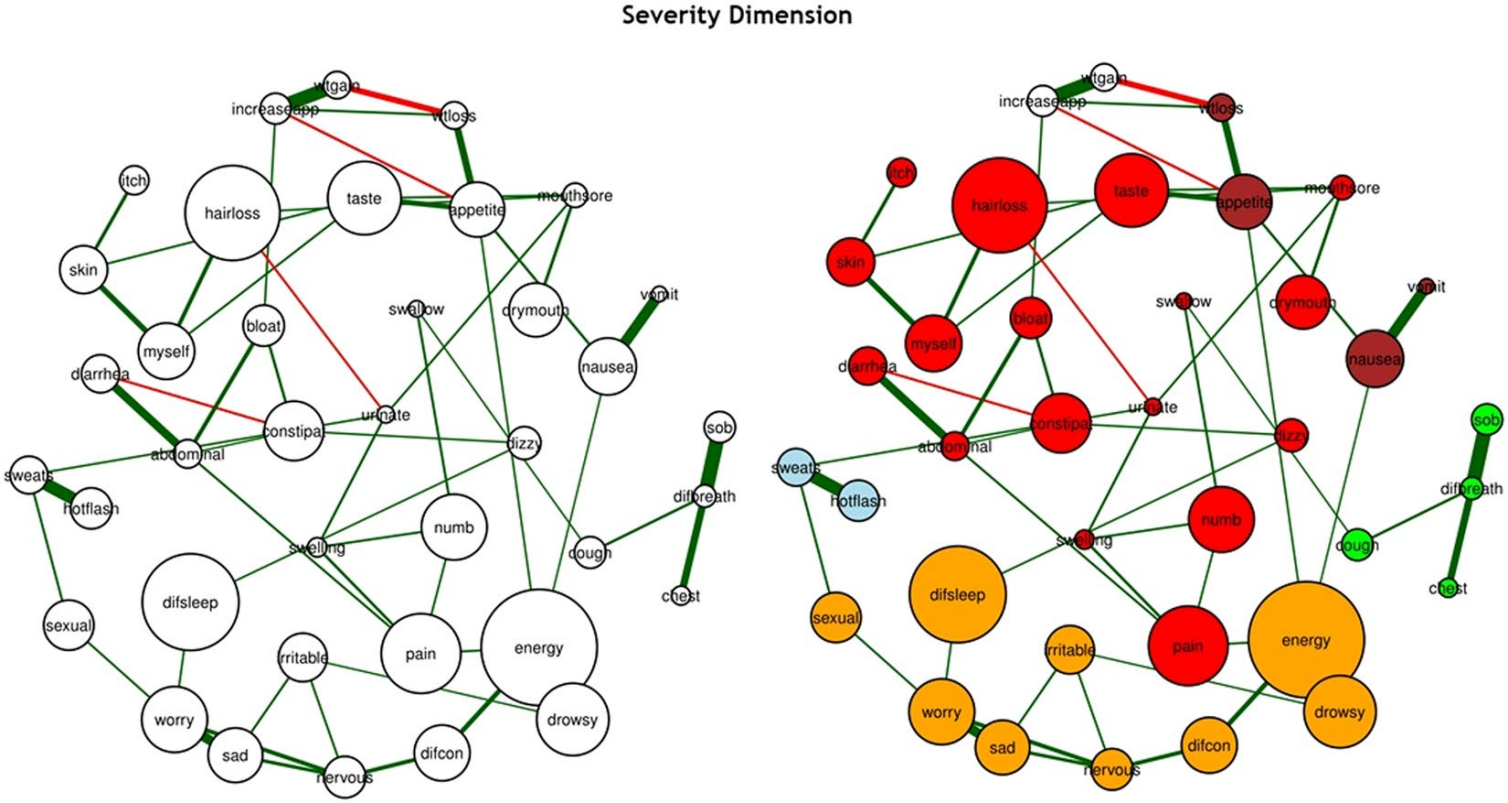
The estimated networks of 38 cancer symptoms across the “severity” dimension (**a**) without the identified communities and (**b**) with the identified communities (walktrap algorithm). Nodes represent symptoms and edges represent a partial correlation between the symptoms, after controlling for all of the other correlations of a given node. The 38 cancer symptoms represented in the nodes above are coded in the following fashion: difcon: Difficulty Concentrating, pain: Pain, energy: Lack of Energy, cough: Cough, nervous: Feeling Nervous, hotflash: Hot Flashes, drymouth: Dry Mouth, nausea: Nausea, drowsy: Feeling Drowsy, numb: Numbness or Tingling in Hands or Feet, chest: Chest Tightness, difbreath: Difficulty Breathing, difsleep: Difficulty Sleeping, bloat: Feeling Bloated, urinate: Problems with Urination, vomit: Vomitting, sob: Shortness of Breath, diarrhea: Diarrhea, sad: Feeling Sad, sweats: Sweats, sexual: Problems with Sexual Interest or Activity, worry: Worrying, itch: Itching, appetite: Lack of Appetite, abdominal: Abdominal Cramps, increaseapp: Increased Appetite, wtgain: Weight Gain, dizzy: Dizziness, swallow: Difficulty Swallowing, irritable: Feeling Irritable, mouthsore: Mouth Sore, wtloss: Weight Loss, hairloss: Hair Loss, constipat: Constipation, swelling: Swelling, taste: Change in the Way Food Tastes, myself: I Do Not Look Like Myself, skin: Changes in Skin.

**Figure 4:**
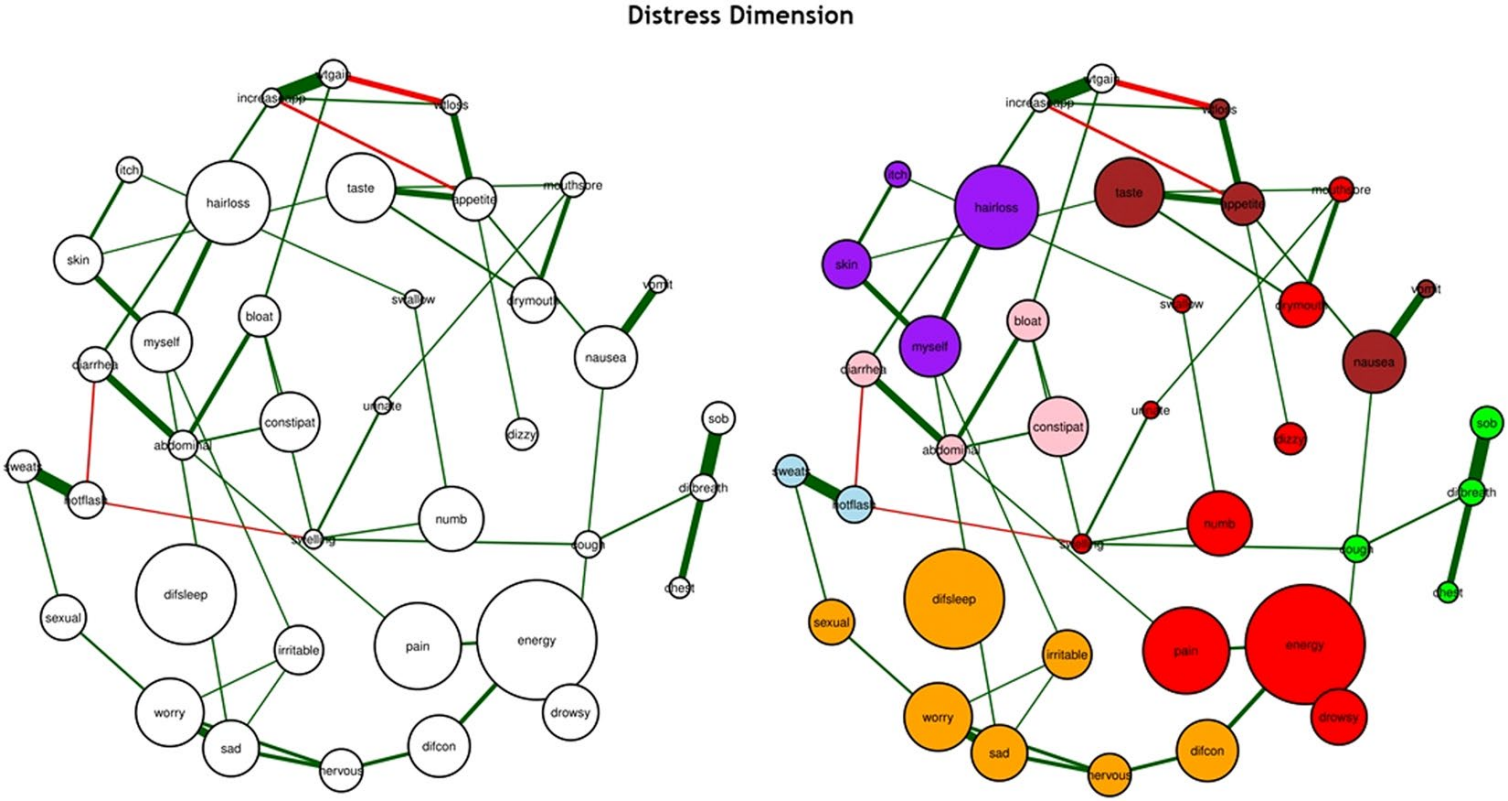
The estimated networks of 38 cancer symptoms across the “distress” dimension (**a**) without the identified communities and (**b**) with the identified communities (walktrap algorithm). Nodes represent symptoms and edges represent a partial correlation between the symptoms, after controlling for all of the other correlations of a given node. The 38 cancer symptoms represented in the nodes above are coded in the following fashion: difcon: Difficulty Concentrating, pain: Pain, energy: Lack of Energy, cough: Cough, nervous: Feeling Nervous, hotflash: Hot Flashes, drymouth: Dry Mouth, nausea: Nausea, drowsy: Feeling Drowsy, numb: Numbness or Tingling in Hands or Feet, chest: Chest Tightness, difbreath: Difficulty Breathing, difsleep: Difficulty Sleeping, bloat: Feeling Bloated, urinate: Problems with Urination, vomit: Vomitting, sob: Shortness of Breath, diarrhea: Diarrhea, sad: Feeling Sad, sweats: Sweats, sexual: Problems with Sexual Interest or Activity, worry: Worrying, itch: Itching, appetite: Lack of Appetite, abdominal: Abdominal Cramps, increaseapp: Increased Appetite, wtgain: Weight Gain, dizzy: Dizziness, swallow: Difficulty Swallowing, irritable: Feeling Irritable, mouthsore: Mouth Sore, wtloss: Weight Loss, hairloss: Hair Loss, constipat: Constipation, swelling: Swelling, taste: Change in the Way Food Tastes, myself: I Do Not Look Like Myself, skin: Changes in Skin.

In network model representations, nodes (symptoms) are represented as circles and links between nodes (edges) are represented as lines (see Figs 2a, 3a and 4a). The size of each node (i.e., symptom) is proportional to the occurrence rate, severity rating, or distress rating of each symptom. Each link in the network represents the interconnections between two symptoms after conditioning on all of the other symptoms in the network. Green lines indicate positive inter-connections. Red lines indicate negative inter-connections. Thicker lines indicate stronger inter-connections. Because the strength of the relationships between symptoms are taken into account, the networks are considered weighted. The layout of these networks is based on the Fruchterman-Reingold algorithm, which estimates the optimal layout so that nodes with stronger and/or more connections are placed closer to each other^43^.

In order to gain additional insights into the structural importance of each node (i.e., symptom) in each of the networks, three centrality indices (i.e., betweenness, closeness, strength) were estimated^28,44^. Nodes with high centrality indices are considered core nodes in the network. *Betweenness* measures the number of times a node lies on the shortest path between two other nodes. This index indicates which nodes may act as bridges between other nodes in the network. *Closeness* summarizes the average distance of a node to all other nodes in the network. Closeness allows for the identification of nodes (i.e., symptoms) that are in a position to have a substantial influence on other node(s) (i.e., other symptom (s)) in the network. *Strength* indicates which node has the strongest overall connections. It is calculated by summing the absolute edge weights that are connected to a specific node. Strength provides a measure for identifying the most connected node (i.e., symptom) inside a network.

Figures S1–S3 in the Appendix illustrate the distribution of each symptom within each dimension (i.e. occurrence, severity, distress). These data are presented to assess whether some of our findings could be due to floor or ceiling effects that affect the properties of our centrality indices^45^.

### Network Accuracy and Stability

Inherent in NA is the problem of obtaining network structures that are sensitive to a specific dataset, or to the specific variables included in a study, and/or the specific estimation methods used. As recommended in the literature^38^, we used bootstrap confidence regions to examine the certainty of the edges and tested for significance between edge weights with *α* = 0.05 based on 1000 bootstrap iterations. To estimate the stability of the order of the centrality indices, we used a case- and node-dropping sub-setting bootstrap technique together with the *correlation stability coefficient* (Cs-coefficient), which is an index of the stability of the centrality indices. The Cs-coefficient quantifies the maximum proportion of cases or nodes, respectively, can be dropped at random to retain, with 95% certainty, a correlation of at least 0.7 with the centralities of the original network^38^. While no strict cut-off value exists for the CS-coefficient, its value should be at least 0.25 and preferably higher than 0.5.

Additionally to the aforementioned analyses, we tested the stability of the centrality indices on four equally divided and randomly assigned subsets. This analysis showed the stability of the identified networks as well as the repeatability of the NA approach on cancer symptoms’ dimensions.

In order to determine whether and how symptoms clustered together inside our networks, we used the Walktrap algorithm^46,47^. The Walktrap algorithm identifies communities (i.e., clusters) of nodes (i.e., symptoms) that are relatively highly connected with each other. Nodes in a community are more likely to connect to other nodes in the same community than to nodes in other communities. Each community corresponds to a connected subgraph. In Figs 2b, 3b and 4b, these communities (i.e., symptom clusters) are visualized with different colors.

## Results

Sample Characteristics - Of the 1328 patients in this study, 77.7% were female and their mean age was 57.2 (±12.4) years. The majority of the patients had breast (40.2%) or gastrointestinal (30.7%) cancer. These patients reported an average of 13.9 (±7.2) symptoms prior to their next dose of CTX. Additional sample characteristics are summarized in Table S1 in the Appendix.

### Network Models of Symptom Occurrence, Severity, and Distress

#### Occurrence

For the occurrence dimension, created using the IsingFit method (see Fig. 2a), we used a gamma value of 0.25 and the OR rule for the nodewise estimation. All of the symptoms were directly or indirectly associated with the network and the network had a medium density (i.e., 36.42% of the potential connections were observed in the network). All connections were positive except for weight gain (wtgain) and weight loss (wtloss).

#### Severity

For the severity dimension, created using the polychoric correlation method and the glasso algorithm (Fig. 3a), we used a tuning parameter of 0.25. All of the symptoms were directly or indirectly associated with the network and the network had a medium density (i.e., 54.48% of the potential connections were observed in the network). All of the connections were positive except for: increased appetite (increaseapp) and lack of appetite (appetite); hair loss (hairloss) and difficulty with urination (urinate); and diarrhea (diarrhea) and constipation (constipat).

#### Distress

For the distress dimension, created using the polychoric correlation method and the Glasso algorithm (Fig. 4a), we used a tuning parameter of 0.25. All of the symptoms were directly or indirectly associated with the network and the network had a medium density (i.e., 50.92% of the potential connections were observed in the network). All of the connections were positive except for: increased appetite (increaseapp) and lack of appetite (appetite); weight gain (wtgain) and weight loss (wtloss); diarrhea (diarrhea) and hot flashes (hotflash); and hot flashes and swelling of the arms and legs (swelling).

**Figure 5:**
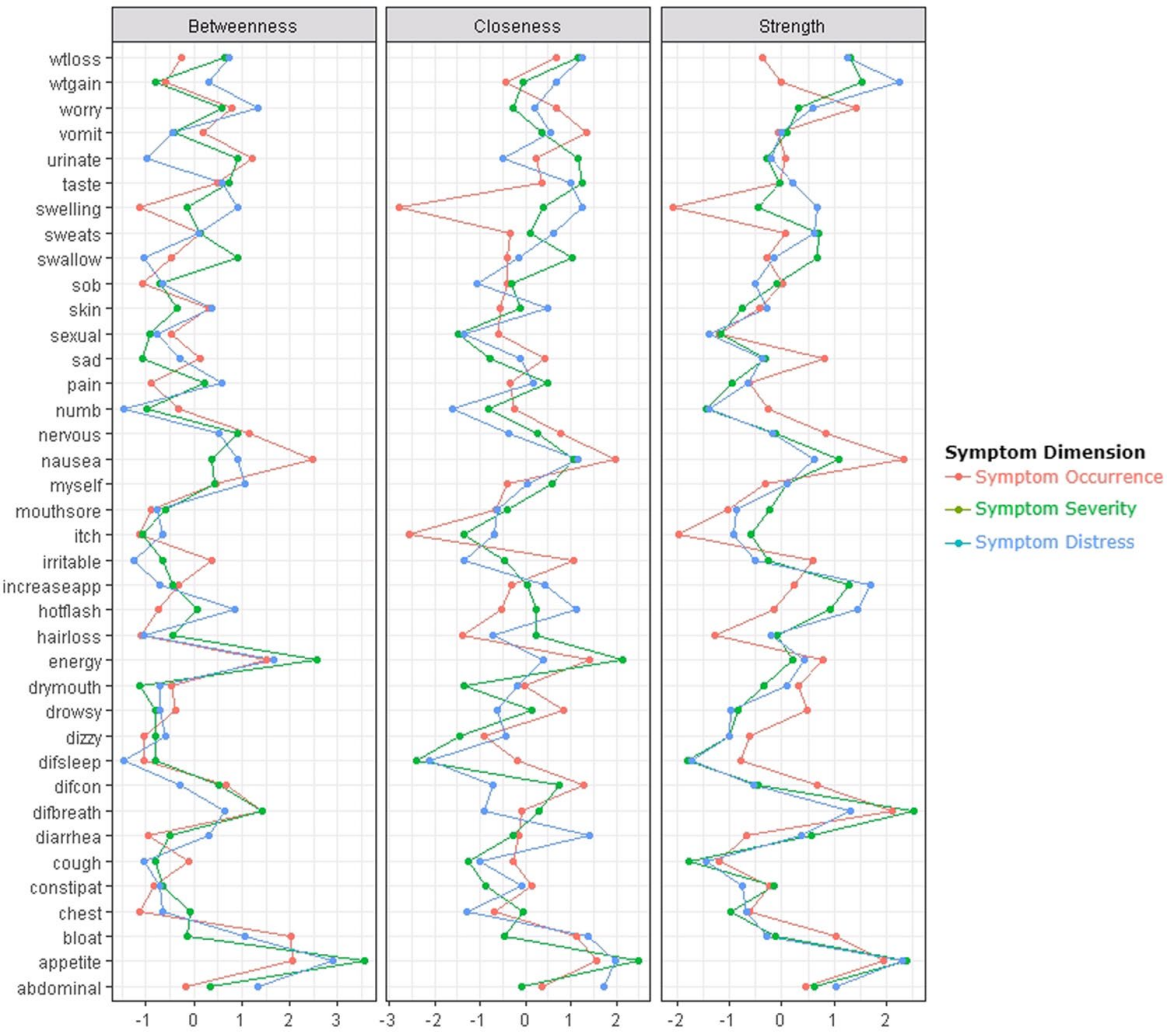
Centrality indices for the estimated network of 38 cancer symptoms shown in Figs 2a to 4a.

To inspect the statistical importance and possible role of each symptom inside each of the the networks, we calculated their centrality indices (Fig. 5). As shown in Supplemental Table S2 in the Appendix, for the symptom occurrence network, nausea and lack of appetite had the highest scores for all three centrality indices. For the severity network, lack of appetite had the highest scores for all three centrality indices and lack of energy had the highest scores across two centrality indices (betweenness and closeness). For the distress dimension, lack of appetite had the highest scores across all three centrality indices.

### Network Accuracy and Stability

Bootstrap confidence regions for the edges’ weights were mostly overlapping (shown in Appendix Fig. S4). The results of the case- and node-dropping bootstrap techniques that were used to estimate the stability of the centrality indices are shown in Appendix Fig. S5. Robustness analyses of the centrality indices showed the following CS-coefficients for each dimension: 1) Occurrence: 0.517 for strength, 0.128 for closeness, and 0.128 for betweenness; 2) Severity: 0.361 for strength, 0.05 for closeness, and 0.284 for betweenness; and 3) Distress: 0.361 for strength, 0.205 for closeness, and 0.128 for betweenness. Across the three symptom dimensions, node strength was the most reliable centrality index.

We also obtained similar results for the node strength for the 4 equally divided and randomly assigned subsets of patients, for each symptom dimension (i.e. occurrence, severity, distress) (See Appendix Figs S6 and S7).

### Communities Within Each Symptom Dimension Network

#### Occurrence

Using the walktrap algorithm (Fig. 2b), the symptoms appear to group into six main clusters: psychological symptom cluster [shown in gold], hormonal symptom cluster [shown in blue], respiratory symptom cluster [shown in green], nutritional symptom cluster [shown in white, yellow, and brown], CTX-related symptom cluster [shown in red], and pain and abdominal symptom cluster [shown in purple].

#### Severity

Using the walktrap algorithm (Fig. 3b), the symptoms appear to group into five main clusters: psychological symptom cluster [shown in gold], hormonal symptom cluster [shown in blue], respiratory symptom cluster [shown in green], nutritional symptom cluster [shown in white and brown], and CTX-related symptom cluster [shown in red].

#### Distress

Using the walktrap algorithm (Fig. 4b), the symptoms appear to group into seven main clusters: psychological symptom cluster [shown in gold], hormonal symptom cluster [shown in blue], respiratory symptom cluster [shown in green], nutritional symptom cluster [shown in white and brown], CTX-related symptom cluster [shown in red], GI symptom cluster [shown in pink], and epithelial symptom cluster [shown in purple].

It should be noted, in the communities (i.e., symptom clusters) that were constructed using the walktrap algorithm, while a number of the symptom clusters have the same names, the specific symptoms within each of these clusters vary across the three dimensions (Table 1).

**Table 1:** Symptom Clusters Derived From Network Analyses of Occurrence, Severity, and Distress.

Symptom Cluster	Occurrence	Severity	Distress
*Psychological Symptom Cluster*	difficulty sleeping	difficulty sleeping	difficulty sleeping
	worrying	worrying	worrying
	feeling sad	feeling sad	feeling sad
	feeling irritable	feeling irritable	feeling irritable
	feeling nervous	feeling nervous	feeling nervous
	difficulty concentrating	difficulty concentrating	difficulty concentrating
	lack of energy	lack of energy	
	feeling drowsy	feeling drowsy	
		problems with sexual interest/activity	problems with sexual interest/activity
*Hormonal Symptom Cluster*	sweats	sweats	sweats
	hot flashes	hot flashes	hot flashes
	problems with sexual interest/activity		
*Respiratory Symptom Cluster*	shortness of breath	shortness of breath	shortness of breath
	difficulty breathing	difficulty breathing	difficulty breathing
	cough	cough	cough
	chest tightness	chest tightness	chest tightness
*Nutritional Symptom Cluster*	weight gain	weight gain	weight gain
	weight loss	weight loss	weight loss
	increased appetite	increased appetite	increased appetite
		nausea	nausea
		vomiting	vomiting
		lack of appetite	lack of appetite
			change in way food tastes
*Chemotherapy-related Symptom Cluster*	itching	itching	
	hair loss	hair loss	
	changes in skin	changes in skin	
	I don’t look like myself	I don’t look like myself	
	change in way food tastes	change in way food tastes	
	lack of appetite		
	mouth sores	mouth sores	mouth sores
	difficulty swallowing	difficulty swallowing	difficulty swallowing
	dry mouth	dry mouth	dry mouth
	vomiting		
	nausea		
	dizziness	dizziness	dizziness
	constipation	constipation	
		swelling of arms or legs	swelling of arms or legs
		problems with urination	problems with urination
		diarrhea	
		abdominal cramps	
		numbness/tingling in hands/feet	numbness/tingling in hands/feet
		pain	Pain
		feeling bloated	
			lack of energy
			feeling drowsy
*Pain and Abdominal Symptom Cluster*	diarrhea	Not identified	Not identified
	abdominal cramps		
	feeling bloated		
	swelling of arms or legs		
	pain		
	numbness/tingling in hands/feet		
	problems with urination		
*Gastrointestinal Symptom Cluster*	Not identified	Not identified	diarrhea
			abdominal cramps
			constipation
			feeling bloated
*Epithelial Symptom Cluster*	Not identified	Not identified	hair loss
			I don’t look like myself
			itching
			skin changes

## Discussion

This study is the first to use NA methods to examine the relationships among 38 common symptoms in a large sample of oncology patients undergoing CTX using ratings of occurrence, severity, and distress. The use of NA to understand the symptom experience of oncology patients has the potential to increase our knowledge of the structural relationships among co-occurring symptoms and symptom clusters; the core symptoms driving associations between and among symptoms, and how co-occurring symptoms and symptom clusters change based on the dimension of the symptom experience that is used to create the network.

Our hypothesis that the network structure for the distress dimension would differ from the occurrence and severity dimensions was partially supported based on visual inspection of the network structures and the larger number of symptom clusters identified in the distress network. For over four decades, emphasis has been placed on an evaluation of multiple dimensions of the symptom experience because each dimension provides distinct and useful information^30–33,48,49^. Occurrence data are used to identify the most common symptoms in oncology patients. Severity data are used to determine the magnitude of a specific symptom and to guide treatment decisions. An evaluation of symptom distress provides information on “the physical or mental anguish or suffering” associated with a symptom^48^. While symptom theory^50–53^ and data from studies that used the MSAS suggest that these three dimensions are distinct^32,33,54–56^, findings from our study provide the first direct evidence that the connections between and among symptoms differ depending on the symptom dimension that was used to create the network.

Because oncology patients experience an average of fifteen unrelieved symptoms that are highly variable in their occurrence, severity, and distress^1–3^, an equally important question in symptom research is to determine which symptom or symptoms is driving the other symptoms. While our NA of cross-sectional data does not demonstrate causality, the centrality indices provide some insights into the structural importance of each of the symptoms within each of the networks. In terms of the occurrence network, nausea had the highest scores for all three centrality indices. In this sample, 47.48% of patients reported nausea prior to their next dose of CTX. While vomiting is well controlled with newer antiemetic regimens, nausea is a persistent symptom that compromises a patient’s nutritional status, results in significant psychological distress, has a negative impact on quality of life, and can result in the discontinuation of cancer treatment^57–59^. For both the severity and distress networks, lack of appetite had the highest scores for all three centrality indices and it was the symptom with the second highest centrality scores for the occurrence dimension. While this symptom was reported by 41.31% of the patients in this study, it is a symptom that is not routinely assessed in oncology patients undergoing cancer treatment. Based on network theory^19,60–63^, given their high centrality index scores, these symptoms may be targets for therapeutic interventions that if successful would reduce other symptoms in the network.

While a tremendous amount of research has focused on the evaluation of symptom clusters in oncology patients^4,5^, our study is the first to use NA to visualize how one symptom cluster is associated with other symptom clusters. To date, the majority of the work to create symptom clusters was done using cluster analysis or factor analysis. While these approaches identified some of the most common symptom clusters in oncology patients, these symptom clusters are created as independent “factors”. Our NA represents a major breakthrough in symptom cluster research. Within each dimension, our graphical representation allows us to visualize how the various symptom clusters within the network are inter-connected with other symptom clusters in the same network. Based on network theory^60,64,65^, we can hypothesize that symptoms on the edges of each of the clusters may have an influence on that cluster. For example, in Fig. 2b, difficulty sleeping and hot flashes are on the edges of their respective symptom clusters. While we cannot demonstrate causality, it is known that the occurrence of hot flashes disrupts patients’ sleep^66,67^. If our findings are confirmed in an independent sample, future NAs can evaluate for causality and test interventions to reduce symptoms across clusters.

In terms of the specific symptom clusters identified for each of the symptom dimensions, our finding of a psychological symptom cluster across all three dimensions is consistent with findings from a recent review that noted that this cluster is one of the most common clusters identified in oncology patients^4^. The other four symptom clusters that were common across all three symptom dimensions (i.e., hormonal, respiratory, nutrition, and CTX-related) were reported in previous symptom cluster studies^68–72^. The fact that two additional and unique symptom clusters were identified within the distress network provide additional support for the hypothesis that symptom distress is a distinct dimension of the oncology patients’ symptom experience. Future research will need to evaluate causality among symptoms within each of the dimension networks and whether common or distinct interventions are needed to decrease the severity and distress associated with a specific symptom.

### Limitations and Future Directions

Several limitations warrant consideration. While our sample was rather large in comparison to the number of parameters estimated, the heterogeneity introduced by the specific demographic and clinical characteristics of the patients in this study may influence the stability of our estimated networks. Since this study is the first to use NA to examine the relationships among co-occurring symptoms and symptom clusters, our findings warrant replication in an independent sample of oncology patients undergoing CTX. In addition, this analysis of cross-sectional data does not allow for causal inferences on the role of each symptom within each of our networks. Finally, because no standards exist to interpret the significance and robustness of networks and because the validity of the visual interpretation of complex networks is subjective, additional research is warranted to confirm our findings.

In terms of directions for future research, our findings warrant replication in an independent sample with similar demographic and clinical characteristics. In addition, comparisons of network structures need to be done among different cancer diagnoses, across different stages of disease, and among different cancer treatments. The impact of various demographic (e.g., age, gender) and clinical (e.g., comorbid conditions, functional status) characteristics on the network structure of cancer symptoms warrants evaluation. Using longitudinal data, NA will allow us to explore the causal relationships among co-occurring symptoms and symptom clusters^12^.

## Conclusion

In this study, we used NA to investigate the relationships among 38 common symptoms in oncology patients receiving CTX. As the first NA of cancer symptoms, our work provides new insights into the inter-relationships among co-occurring symptoms and symptom clusters. Findings from this study suggest that the connections between and among symptoms may differ depending on the symptom dimension used to create the network. Our findings suggest that distress may be a different dimension of a patient’s symptom experience. In addition, this study provides the first visualizations of the inter-relationships among symptom clusters across three dimensions of the patients’ symptom experience. While these findings warrant confirmation in an independent sample, we believe that NA has the potential to improve our understanding of the oncology patients’ symptom experience so that individualized and targeted interventions can be prescribed to reduce each patient’s symptom burden.

## Supplementary information

Appendix in pdf format

## Data Availability

The data used in this study will be available upon request and subject to ethics approval. All data requests should be sent to Christine Miaskowski (chris.miaskowski@ucsf.edu).
