# Supplementary material for: Network Analysis of the Multidimensional Symptom Experience of Oncology
Source: Sci Rep. 2019 Feb 19;9:2258. doi: 10.1038/s41598-018-36973-1 (PMC6381090; doi:10.1038/s41598-018-36973-1)
Supplement: Supplementary file 1 — Appendix in pdf format [file 41598_2018_36973_MOESM1_ESM.pdf]

Network Analysis of the Multidimensional Symptom Experience of Oncology

Nikolaos Papachristou[1,*+], Payam Barnaghi[1+], Bruce Cooper[2], Kord M. Kober[2], Roma Maguire[3], Steven M. Paul[2], Marilyn Hammer[4], Fay Wright[5], Jo Armes[1], Eileen P.Furlong[6], Lisa McCann[3], Yvette P. Conley[7], Elisabeth Patiraki[8], Stylianos Katsaragakis[9], Jon D. Levine[2], and Christine Miaskowski[2]

[1]University of Surrey, Guildford, UK
[2]University of California, San Francisco, USA
[3]University of Strathclyde, Glasgow, Scotland
[4]Department of Nursing, Mount Sinai Medical Center, New York, USA
[5]School of Nursing, Yale University, New Haven, USA
[6]School of Nursing, Midwifery and Health Systems, University College Dublin, Dublin, Ireland
[7]School of Nursing, University of Pittsburgh, Pittsburgh, USA
[8]National and Kapodistrian University of Athens, Athens, Greece
[9]Faculty of Nursing, University of Peloponnese, Sparti, Greece
[*]n.papachristou@surrey.ac.uk
[+]these authors contributed equally to this work

Supplemental Tables S1-S2

Table S1. Demographic and Clinical Characteristics of the Sample (n=1328)

Characteristic	Mean (SD)
Age (years)	57.2 (12.4)
Education (years)	16.2 (3.0)
Body mass index (kg/m2)	26.2 (5.7)
Karnofsky Performance Status score	80.0 (12.5)
Self-administered Comorbidity Questionnaire score	5.5 (3.2)
Time since diagnosis (years)	2.0 (3.9)
Time since diagnosis (years, median)	0.42
Number of prior cancer treatments	1.6 (1.5)
Number of metastatic sites including lymph node involvement[a]	1.2 (1.2)
Number of metastatic sites excluding lymph node involvement	0.8 (1.1)
	**% (n)**
Gender (% female)	77.7 (1044)
Self-reported ethnicity	
White	69.5 (921)
Non-white	30.5 (405)
Married or partnered (% yes)	64.5 (854)
Lives alone (% yes)	21.4 (284)
Currently employed (% yes)	35.1 (466)
Annual household income	
Less than $30,000	18.4 (221)
$30,000 to $70,000	21.1 (254)
$70,000 to $100,000	16.9 (203)

Continued on next page

Table S1 – continued from previous page

	% (n)
Greater than $100,000	43.5 (523)
Child care responsibilities (% yes)	22.1 (290)
Elder care responsibilities (% yes)	8.0 (97)
Exercise on a regular basis (% yes)	70.8 (929)
Cancer diagnosis	
Breast cancer	40.2 (540)
Gastrointestinal cancer	30.7 (412)
Gynecological cancer	17.3 (233)
Lung cancer	11.8 (158)
Prior cancer treatment	
No prior treatment	24.9 (325)
Only surgery, CTX, or RT	42.1 (549)
Surgery and CTX, or surgery and RT, or CTX and RT	19.8 (259)
Surgery and CTX and RT	13.2 (172)
Metastatic sites	
No metastasis	32.3 (428)
Only lymph node metastasis	22.1 (292)
Only metastatic disease in other sites	21.1 (280)
Metastatic disease in lymph nodes and other sites	24.5 (324)
Cycle length	
14 day cycle	42.2 (565)
21 day cycle	50.6 (678)
28 day cycle	7.2 (97)

[a] Total number of metastatic sites evaluated was 9.
Abbreviations: CTX = chemotherapy, kg = kilograms, m^2 = meters squared, RT = radiation therapy, SD = standard deviation

Table S2. Summary of Centrality Measures for Each of the Symptom Dimensions

Symptom	Betweenness	Rank	Closeness	Rank	Strength	Rank
Occurrence						
nausea	2.47	1	1.97	1	2.35	1
lack of appetite	2.07	2	1.56	2	1.94	3
feeling bloated	2.03	3	1.11		1.04	5
lack of energy	1.5	4	1.41	3	0.8	
difficulty breathing	1.44	5	-0.09		2.12	2
worrying	0.8		0.69		1.41	4
vomiting	0.2		1.36	4	-0.07	
difficulty concentrating	0.67		1.29	5	0.68	
Severity						
lack of appetite	3.55	1	2.49	1	2.38	2
lack of energy	2.56	2	2.15	2	0.22	
difficulty breathing	1.42	3	0.31		2.52	1
difficulty with urination	0.9	4	1.15	4	-0.3	
difficulty swallowing	0.9	4	1.02		0.67	
weight loss	0.64		1.15	5	1.31	3
increased appetite	-0.45		0.05		1.28	4
change in way food tastes	0.75		1.23	3	-0.04	
nausea	0.38		1.07		1.1	5

Continued on next page

Table S2 – continued from previous page

Symptom	Betweenness	Rank	Closeness	Rank	Strength	Rank
Distress						
lack of appetite	2.9	1	1.97	1	2.31	1
lack of energy	1.67	2	0.38		0.42	
worrying	1.33	3	0.21		0.61	
abdominal cramps	1.33	4	1.71	2	1.04	
I don't look like myself	1.06	5	0.06		0.09	
feeling bloated	1.06	5	1.36	4	-0.28	
diarrhea	0.31		1.4	3	0.37	
weight loss	0.72		1.25	5	1.25	5
weight gain	0.31		0.67		2.25	2
increased appetite	-0.07		0.41		1.71	3
hot flashes	0.86		1.11		1.44	4

Supplemental Figures S1-S5

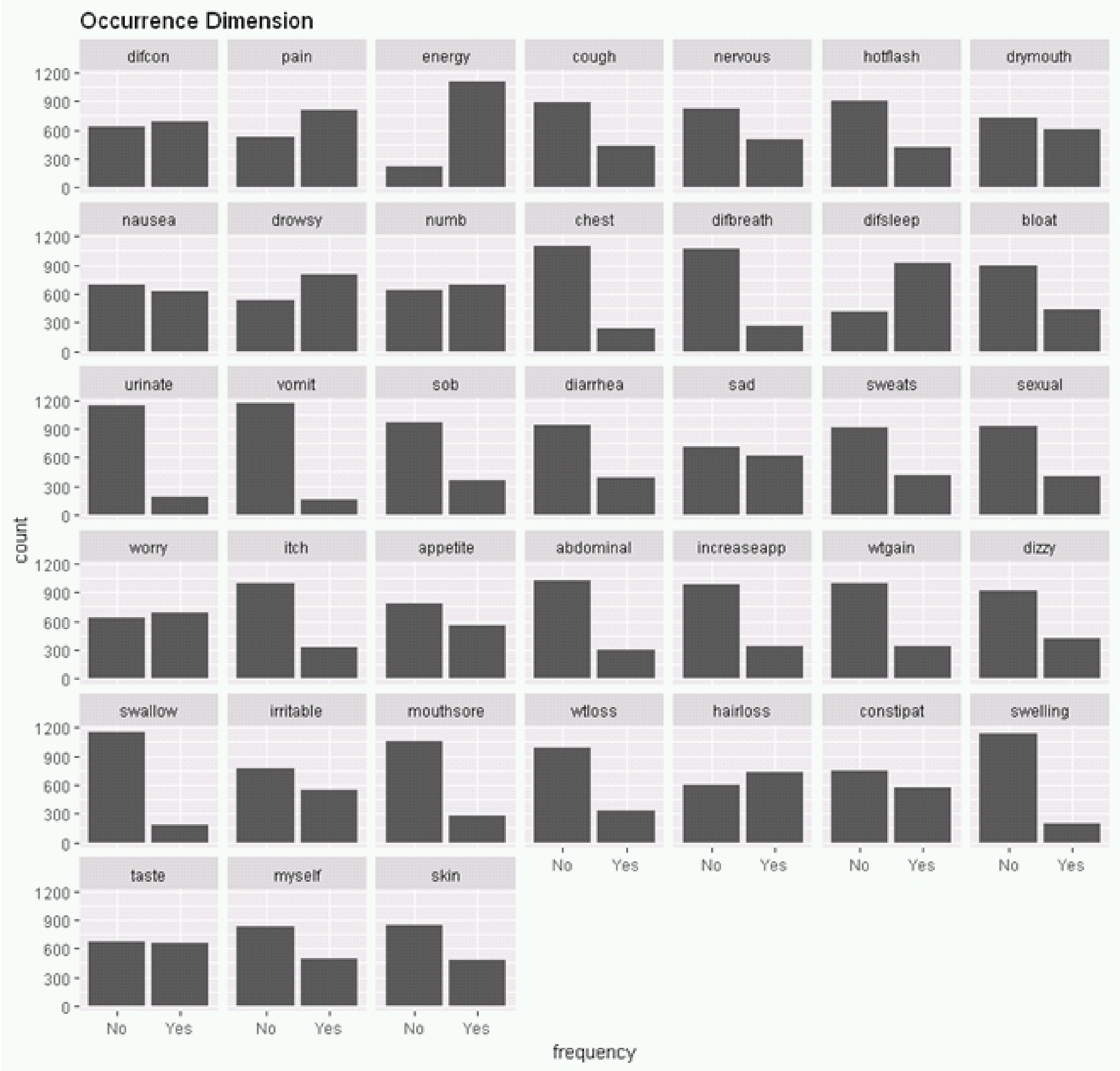

Figure S1. Frequency of the 38 Symptoms (Occurrence Dimension)

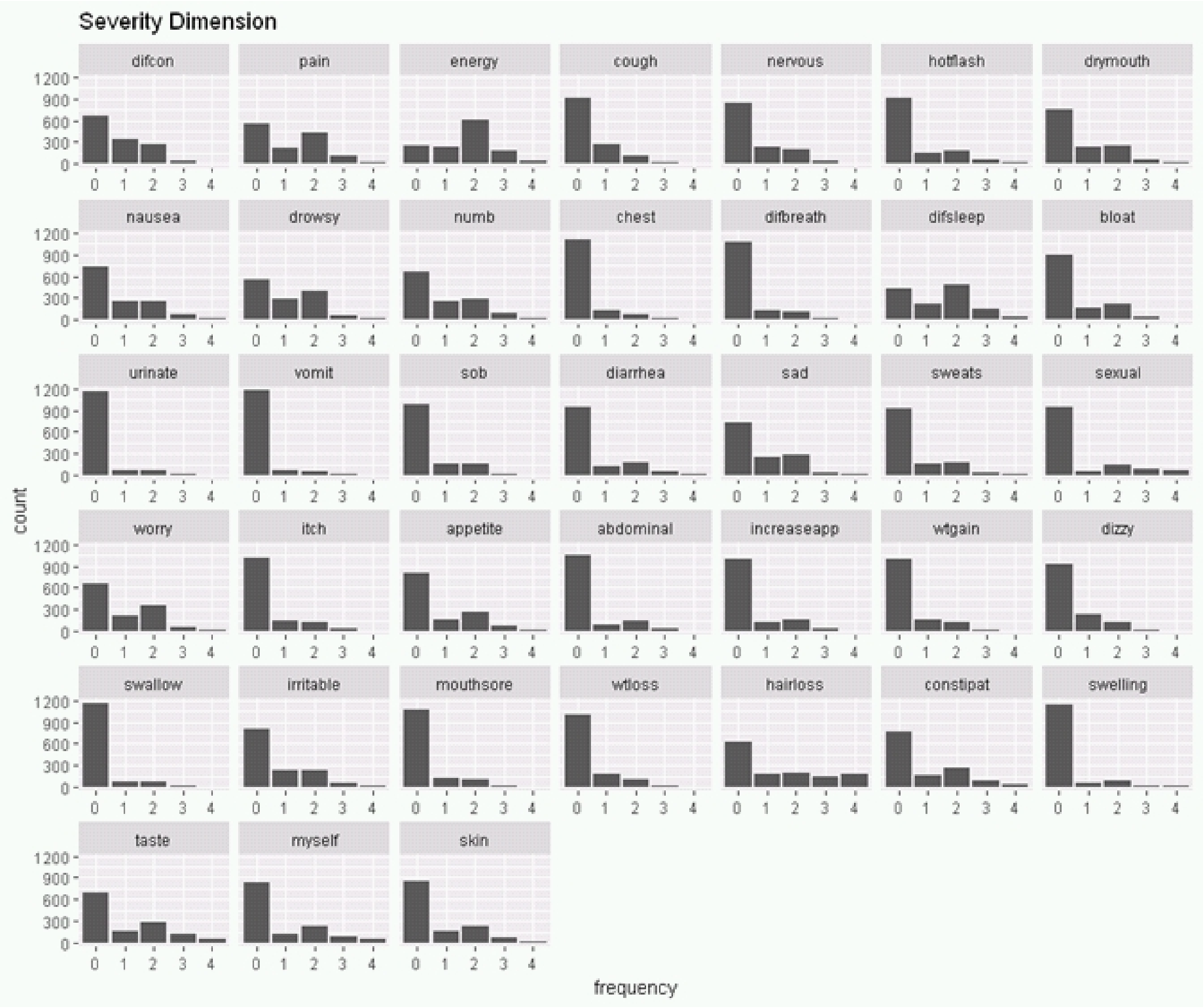

Figure S2. Frequency of the 38 Symptoms (Severity Dimension)

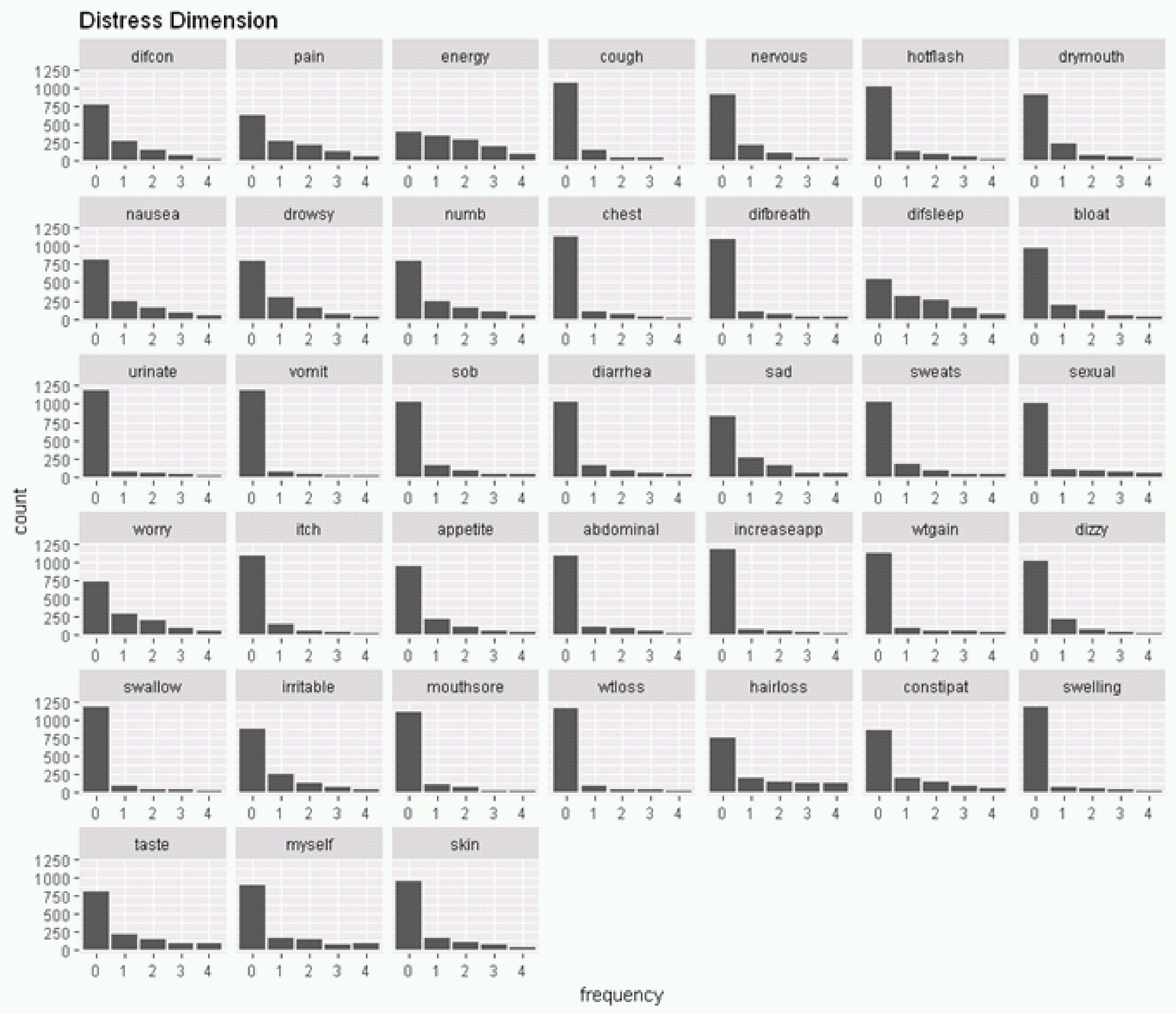

Figure S3. Frequency of the 38 Symptoms (Distress Dimension)

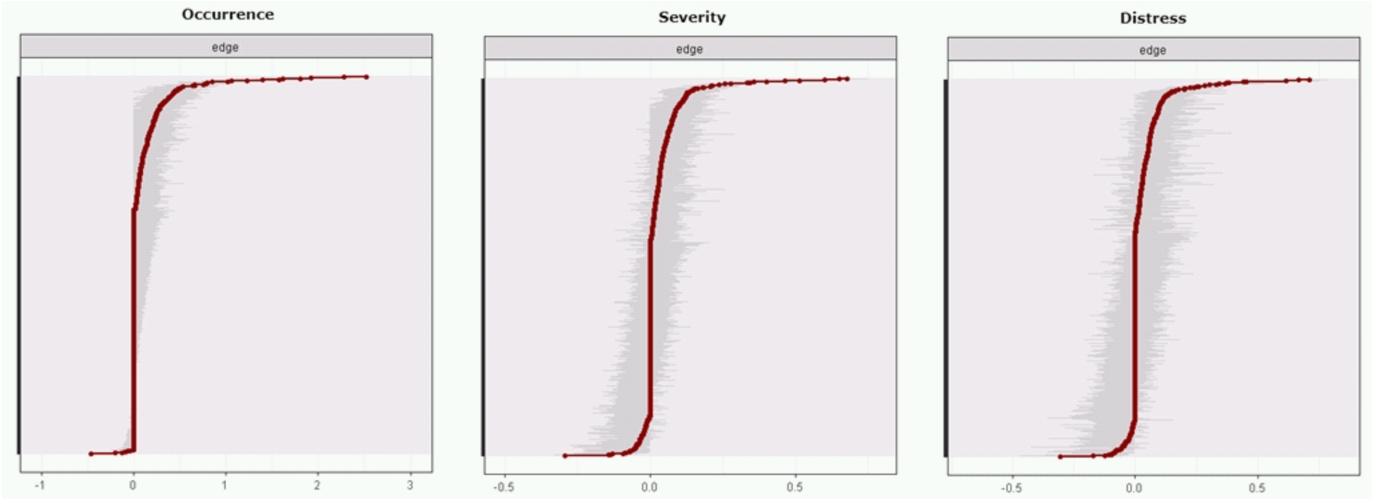

Figure S4. Bootstrap 95% confidence intervals for estimated edge weights for the network of the 38 cancer symptoms across the three dimensions (i.e. occurrence, severity, distress). The edge weights, each horizontal line representing one edge, are represented by the red line, the 95% confidence intervals by the grey area.

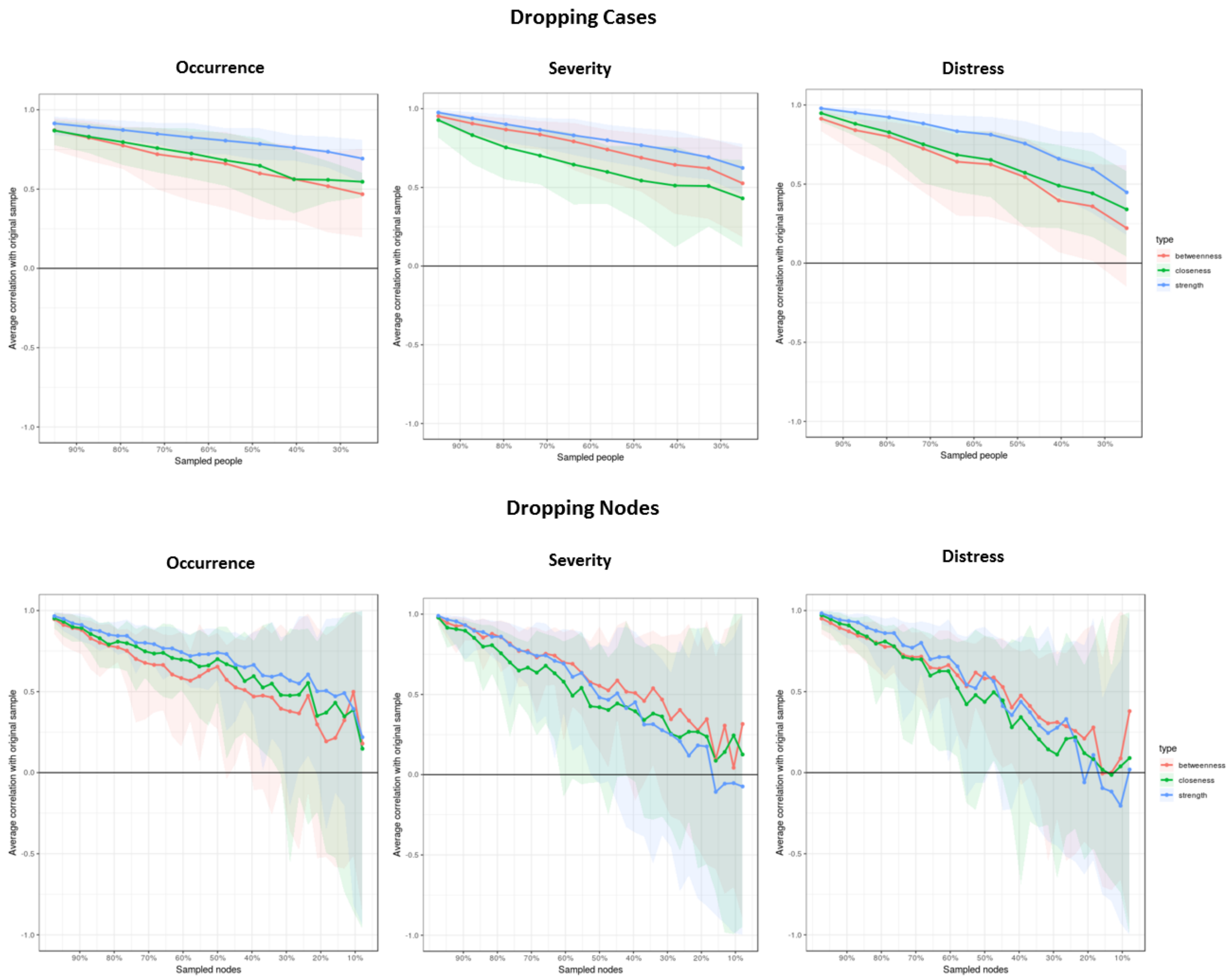

Figure S5. Average correlations between the centrality indices of the networks of the 38 cancer symptoms across the three dimensions (i.e. occurrence, severity, distress) sampled with cases or nodes dropped and the original sample. Lines indicate the means and areas indicate the range from the 2.5th quantile to the 97.5th quantile.

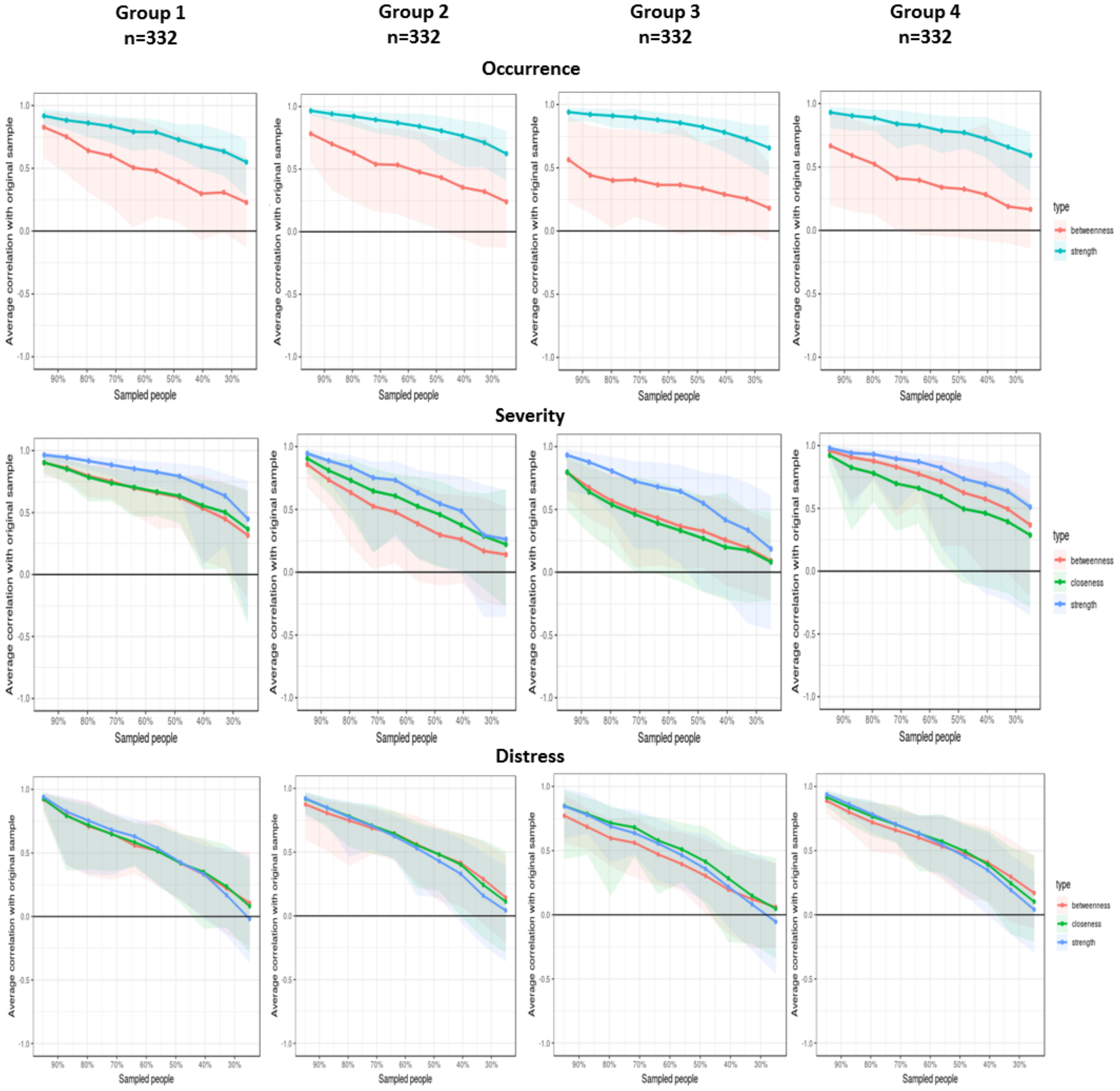

Figure S6. Average correlations between the centrality indices of the networks of the 38 cancer symptoms in each of **4 equally divided groups**, across each dimension (i.e. occurrence, severity, distress) sampled **with cases dropped** and the original sample. Lines indicate the means and areas indicate the range from the 2.5th quantile to the 97.5th quantile. Across the symptom dimension of Occurrence, the statistic Closeness does not contain any variance and is therefore not shown.

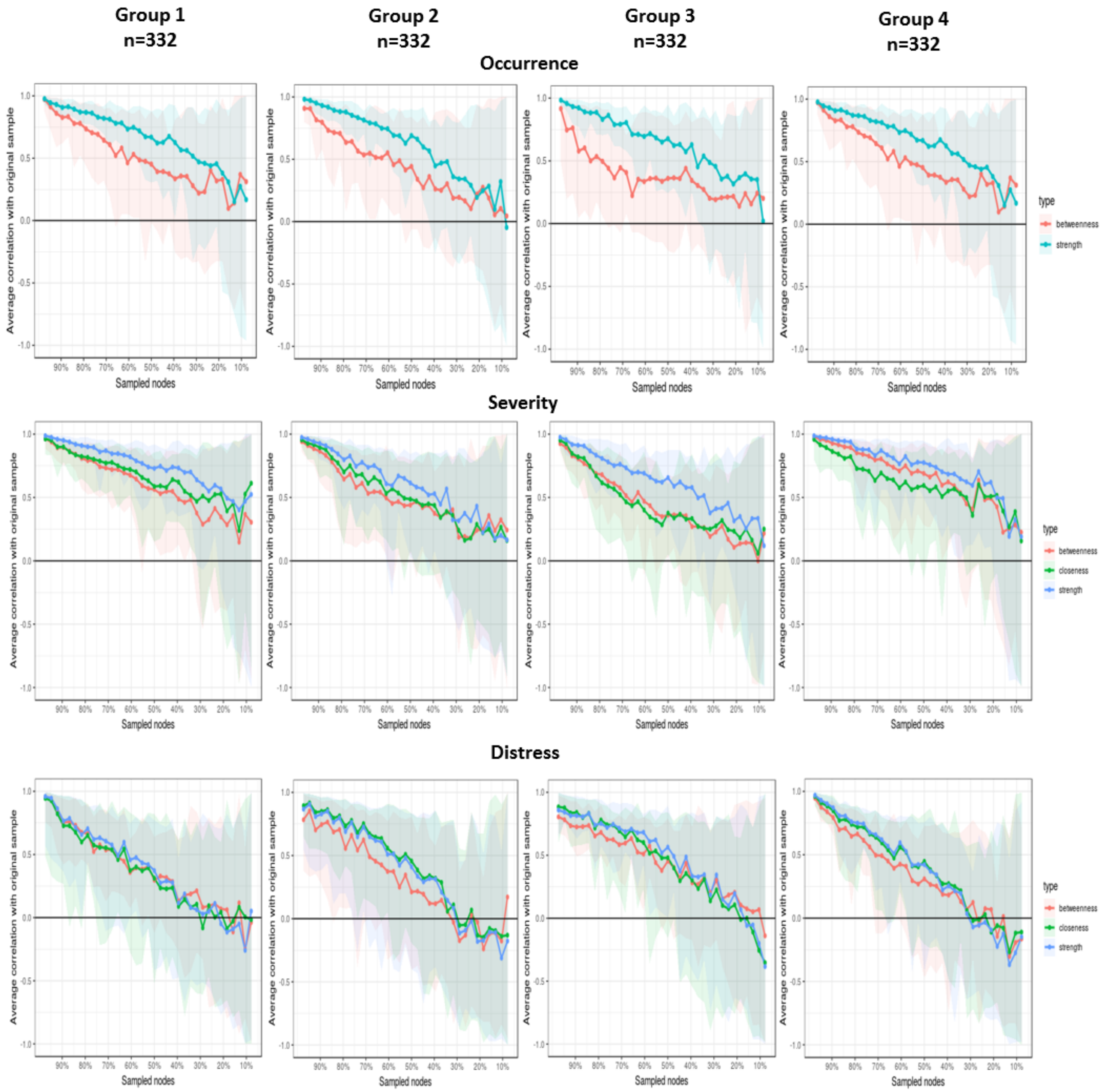

Figure S7. Average correlations between the centrality indices of the networks of the 38 cancer symptoms in each of **4 equally divided groups**, across each dimension (i.e. occurrence, severity, distress) sampled **with nodes dropped** and the original sample. Lines indicate the means and areas indicate the range from the 2.5th quantile to the 97.5th quantile. Across the symptom dimension of Occurrence, the statistic Closeness does not contain any variance and is therefore not shown.
